# Living Within and Without: Life-Space Mapping to Visualize Need and Resource Access of Rural Dementia Dyads

**DOI:** 10.1093/geroni/igaa057.1404

**Published:** 2020-12-16

**Authors:** Julia Loup, A Lynn Snow, Michelle Hilgeman

**Affiliations:** 1 University of Alabama, Tuscaloosa, Alabama, United States; 2 Tuscaloosa VA Medical Center, Tuscaloosa, Alabama, United States

## Abstract

Rural-dwelling veterans with dementia (PWD) and their family caregivers (CG) have unique needs and resource access limitations. Life-Space assessment models suggest older adults’ needs are reflected in their daily-life mobility and routines (Peel et al., 2005). Yet, medical treatment models seldom incorporate non-health related activities (e.g., transportation, groceries, distance to formal and informal support networks). This mixed-methods study proposes an exploratory life-space modeling visualization that integrates qualitative and quantitative daily-life data from rural dwelling dyads in Alabama. Two case studies are selected from a sample of 30 qualitative interviews to demonstrate this innovative analytic approach. One case depicts a married dyad (PWD and spousal CG) (CGage = 74; PWDage = 80, PWD MoCA score = 21) and the second visualization is of a PWD living alone (PWDage = 82, PWD MoCA Score = 20). Daily-life experiences and routines mentioned during interviews were categorized using a rapid analysis template approach and informed by unmet needs theories (Algase et al., 1996). Next, extracted data were placed into mapping visualization software. The maps include visual cues (colors, transportation routes, and icons) to designate met, unmet, and vulnerable needs and resources, allowing visual interaction with the two cases’ dementia caregiving context and qualitative responses. Life-space maps may be useful tools to visualize resource access and assist integrated health care systems in better understanding daily interactions and intervention gaps for difficult to reach populations. Future developments include ecological momentary assessment and Global Positioning System (GPS) data to develop life-space maps using real-time data collection.

